# Quantitative genetic analysis of diamide resistance in Australian *Spodoptera litura* (Fabricius) (Lepidoptera: Noctuidae)

**DOI:** 10.1038/s41598-025-12292-0

**Published:** 2025-07-27

**Authors:** L. J. Bird, N. M. Dunn

**Affiliations:** NSW Department of Primary Industries, Tamworth Agricultural Institute, Marsden Park Rd, Calala, NSW 2340 Australia

**Keywords:** Cluster caterpillar, Chlorantraniliprole, Insecticides, Resistance management, Cross-resistance, Synergists, Heritable quantitative trait, Agroecology

## Abstract

*Spodoptera litura* is a major insect pest of horticulture, cotton and legume crops which has developed resistance to insecticides from many chemical classes. Chlorantraniliprole is an important option for selective control of *S. litura*, and in 2023 a population established from grain legume fields in northwest Western Australia (BM28x) was found to have > 1000-fold resistance to chlorantraniliprole compared with laboratory reference strain (KSL). Following its establishment in the laboratory, the BM28x strain was found to be homozygous for resistance to chlorantraniliprole and subsequent characterisation by quantitative genetic analysis showed that resistance was autosomal, incompletely recessive (*D*_LC_ = 0.185) and conferred by one or a few closely linked loci. Chlorantraniliprole resistance in the BM28x strain conferred major cross-resistance to cyantraniliprole (262-fold) and tetraniliprole (591-fold). However, there was minimal cross-resistance (≤ 3.3-fold) in the BM28x strain to broflanilide, emamectin benzoate, spinetoram, methoxyfenozide and the Vip3A protein expressed by the bacterium *Bacillus thuringiensis*, indicating that resistance could be managed effectively using chemical rotational strategies which incorporate transgenic technologies. Inhibition bioassays using the synergists piperonyl butoxide, triphenyl phosphate and diethyl maleate indicate that metabolic detoxification is not involved in resistance to chlorantraniliprole in the BM28x strain. The implications of high level diamide resistance for management of Australian *S. litura* is discussed.

## Introduction

*Spodoptera litura* (Fabricius) (Lepidoptera: Noctuidae) is a highly polyphagous insect pest with a host range of over 112 species from 40 plant families including high value commodities such as cotton, grain legumes, ornamentals and horticultural crops^1^. Although larvae are primarily leaf feeders, they can also feed on fruiting structures^2^ and at high population densities can cause complete defoliation of plants^1^. *Spodoptera litura* has a wide geographical distribution throughout North Africa, Asia and Australia, and is most prevalent in regions where the climate ranges from tropical to temperate^3^. There is no known adaptive strategy such as diapause for withstanding sub-zero conditions^4^ which is an important limiting factor that largely restricts year-round distribution of *S. litura* to tropical and sub-tropical cropping zones of Australia.

In Australian horticultural and grain legume production, *S. litura* has been incidentally controlled by selective insecticides including spinosyns, avermectins, indoxacarb and diamide insecticides which are used widely to target *Helicoverpa armigera* and *Spodoptera frugiperda*^5^. Widespread use of transgenic cotton (Bollgard^®^3) expressing insecticidal proteins from *B. thuringiensis* (Bt) provides less robust control than for the primary target pest *Helicoverpa* species because, like other Spodopteran species, *S. litura* has a naturally higher tolerance to Cry proteins, with Vip3A being primarily responsible for the lethal toxicity of Bt cotton in *S. litura* with a lesser contribution from Cry2Ab^6^. Potential declines in Bt production under certain environmental conditions may result in outbreaks of *S. litura* in Bollgard 3 crops, requiring applications of synthetic insecticides^7^.

Frequent use of insecticides against this pest in many parts of the world has resulted in field-evolved resistance to broad spectrum insecticides such as organophosphates, carbamates and synthetic pyrethroids as well as to newer active compounds including spinosad, emamectin benzoate, indoxacarb^8^ and diamide insecticides^9,10^. In 2023, routine resistance testing of an *S. litura* population from grain legume crops in northwest Western Australia (WA) showed that 100% of larvae survived exposure to a discriminating dose of chlorantraniliprole which prompted our current investigations into diamide resistance in this species.

Diamide insecticides act selectively on the ryanodine receptor (RyR) in muscle cells of insects causing uncontrolled release of calcium from sarcoplasmic reticulum resulting in impaired regulation of muscle contraction, leading to paralysis and feeding cessation in insects^11^. Resistance to diamides can be caused by two major mechanisms including target site insensitivity and metabolic detoxification. Target site resistance reduces receptor binding on the calcium channel^12^ and is conferred by a small number of highly conserved point mutations in genes which encode for target sites at C-terminal regions of the RyR^13^. The amino acid substitution (G4946E) located close to the C-terminus of the RyR channel was the first mutation to be identified in resistant populations of *Plutella xylostella* from the Philippines and Thailand^14^, with this mutation subsequently found in *P. xylostella* from another ten countries^15^. Homologous point mutations were also identified in populations of *Tuta absoluta* from southern Europe^16^ and *Chilo suppressalis* from China^17^. A second RyR amino acid substitution (I4790M) was detected in populations of *P. xylostella* from China^18^, with homologous point mutations also found in *T. absoluta* (I4746M/T)^16^, *C. suppressalis* (I4758M)^17^ and *Orius laevigatus* (I4790M)^19^. A further point mutation (I4790K) was found in diamide resistant populations of *P. xylostella* from Japan^20^, China^21^, Taiwan^22^ and Australia^23^.

Metabolic detoxification of diamide insecticides is mediated by cytochrome P450, carboxylesterase, and glutathione S-transferase enzymes^24^. The magnitude of metabolic resistance is lower than target site resistance and detoxification pathways appear to be composed of small, additive effects which vary widely between species and are unlikely to produce field failures on their own^13^. Nevertheless, there were several notable studies of chlorantraniliprole resistance in *P. xylostella* from China which showed up-regulation of the cytochrome P450 genes CYP321E1^25^ and CYP6BG1^26^, and up-regulation of a UDP-glycosyltransferases gene (UGT2B17)^27^. In a chlorantraniliprole resistance strain of *C. suppressalis* from China, the P450 enzymes CYP6CV5, CYP9A68, CYP321F3, and CYP324A12 were found to be constitutively overexpressed at a level that produced a resistance ratio of 43-fold compared to a susceptible strain^28,29^ while chlorantraniliprole resistance in another Chinese strain of *C. suppressalis* was largely suppressed by metabolic inhibitors^30^.

Based on the large number of different active ingredients to which Spodopteran pests have developed resistance^31^ and the ongoing reliance on chemical insecticides for their control, there is clearly an urgent need to investigate emerging cases of resistance to pivotal insecticides such as the diamides. Point mutations associated with diamide resistance have been identified in *Spodoptera frugiperda* from Brazil; (I4734M)^32^ and (I4790K)^33^. Widespread diamide use throughout China has also resulted in the emergence of the I4743M mutation in *Spodoptera exigua*^34^ and the I4790M mutation in *S. litura*^10^. However, the role of metabolic resistance to diamides in Spodopteran species seems unclear from studies of *S. exigua*^35^ *and S. litura*^36,37^ and may vary depending on specific insecticides and metabolic inhibitors^38^.

The aim of this study was to characterise resistance in a population of *S. litura* from grain legume fields in northwestern WA which had developed very high levels of insensitivity to chlorantraniliprole. This was achieved by performing a quantitative genetic analysis using specific crosses between the chlorantraniliprole resistant strain, a laboratory reference strain, and the resulting F_1_ hybrid strains. We used bioassays to (1) determine the degree of dominance of resistance, (2) estimate the minimum number of segregating factors involved in inheritance of resistance and the degree to which it was monogenic or polygenic, (3) determine the patterns of cross-resistance by testing the resistant strain with chemical insecticides as well as Bt insecticidal toxins deployed in Australian varieties of transgenic cotton, and (4) investigate the involvement of metabolic enzymes in resistance.

## Materials and methods

### Insect strains

The reference strain of *S. litura* was established from field collections of larvae from cotton crops in the Kununurra region of northern WA (− 15.7787, 128.7438) during 2021 by S. Holmon (Department of Primary Industries, Queensland, Australia). The reference strain, hereafter referred to as the KSL strain, was maintained in the laboratory at Tamworth Agricultural Institute (TAI), New South Wales (NSW), Australia, for four years without exposure to insecticides. The chlorantraniliprole resistant strain of *S. litura* was established from approx. 50 larvae collected from cowpea fields in the Broome region in northwestern WA (− 17.9608, 122.2287) during November 2023. The larvae were reared to moths in the laboratory at TAI and bulk mated to produce F_1_ progeny. The population was reared for a further two generations before being tested for resistance using discriminating dose and dose-response bioassays. Survivors of the discriminating dose of chlorantraniliprole (the concentration known to kill 99.9% of susceptible *S. litura*) were established in a population designated the BM28x strain.

### Insect rearing

Rearing methods were modified from previously described methods^39^. Briefly, egg masses of *S. litura* were collected over four or five days and approx. 100 neonates were transferred to each of ten 250 ml round tubs containing a one-centimetre-thick layer of artificial diet comprised of the following ingredients: 130 g soybean flour, 60 g stabilised wheat germ, 53 g brewers yeast, 31 g agar, 6.1 g ascorbic acid, 3.8 g methyl-hydroxybenzoate and 1.8 g sorbic acid in 1.6 L of water purified by reverse osmosis, hereafter referred to as RO water. Larvae of *S. litura* were bulk-reared in these tubs to the third or fourth instar before being transferred individually into 45-well rearing trays (Tacca Plastics, Moorebank, NSW, Australia) that were covered and heat-sealed with perforated lids (Unipac Solutions, Albion Park, NSW, Australia). When larvae reached the sixth instar they were transferred to 32-well trays (Tacca Plastics, Moorebank, NSW, Australia), covered and heat-sealed with perforated lids (Oliver Products, Grand Rapids, MI, USA). Pupae were removed from trays and placed in 26 cm × 18 cm × 15 cm containers lined with paper towel (Halyard, North Ryde, NSW, Australia) and secured by a tight-fitting lid with a large hole for aeration. Moths were provided with a 4% nectar solution comprising equal parts honey (20 g) and sugar (20 g) dissolved in 1 L of RO water^40^ and fed through a cotton wick. The paper lining the containers provided an oviposition substrate and eggs were collected every two or three days. Insect strains were maintained at 26 ± 1 °C with 14:10 (L: D) h photoperiod and ambient RH throughout their lifecycle.

### Insecticides

Seven commercial insecticide formulations from five mode-of-action (MoA) groups were used in diet incorporation bioassays. These included: three Group 28 insecticides, chlorantraniliprole (Altacor^®^ [35% active ingredient]), cyantraniliprole (Exirel^®^ [10% active ingredient], FMC Australia Ltd., Macquarie Park, NSW, Australia and tetraniliprole (Vayego^®^ [20% active ingredient], Bayer CropScience Pty Ltd, Hawthorn East, VIC, Australia; a meta-diamide insecticide broflanilide (Cimegra^®^ [10% active ingredient]), BASF Australia Ltd., Southbank, VIC, Australia; a Group22A insecticide indoxacarb (Steward^®^ [15% active ingredient]), FMC Australia Ltd., Macquarie Park, NSW, Australia; a Group 6 insecticide emamectin benzoate (Affirm^®^ [1.9% active ingredient]), Syngenta Crop Protection, Macquarie Park, NSW, Australia; a Group 5 insecticide spinetoram (Success Neo^®^ [12% active ingredient]), Corteva Agriscience, Chatswood, NSW, Australia; and a Group 18 insecticide methoxyfenozide (Prodigy^®^ [24% active ingredient]), DuPont Australia Ltd., Macquarie Park, NSW, Australia.

Bioassays to determine cross-resistance to the Bt insecticidal protein Vip3A and expressed by Bollgard^®^3 cotton were performed using Vip3A provided by the Commonwealth Scientific and Industrial Research Organisation (CSIRO), Canberra, ACT, Australia. A Vip3A clone in *Escherichia coli* was used as the source of toxin and the production of quantities for bioassay were based on the procedures of Sena et al.^41^. Briefly, a culture of *E. coli* was grown in Luria-Bertani medium at 37 °C overnight in a shaking incubator. Protein expression was enhanced by adding isopropyl-D-thiogalactopyranoside (IPTG) to the culture and incubating at 28 °C for a further 12 h. Cells were harvested by centrifugation before being resuspended and sonicated in phosphate-buffered saline (PBS). Purified Vip3A protein was prepared from a fraction of the sonicated cell lysate using a HIS SELECT 1 ml cartridge (Sigma Aldrich, Bayswater, VIC, Australia). The resulting preparation of Vip3A protein was examined for purity by electrophoresis (SDS-PAGE) and the concentration of Vip3A was quantified using the Bradford protein assay with BSA as a standard.

Synergism bioassays were performed using the metabolic inhibitors piperonyl butoxide (PBO) (90%), triphenyl phosphate (TPP) (99%), and diethyl maleate (DEM) (96%) to test for involvement of mixed function oxidase, carboxylesterase and glutathione S-transferase, respectively. All synergists were supplied by Sigma-Aldrich (Bayswater, VIC, Australia) and dissolved in analytical grade (99%) acetone (ChemSupply, Port Adelaide, SA, Australia) at a concentration known as the highest dose to cause no mortality in topical bioassays of noctuid larvae (50 µg/µl)^40,42^.

### Insect bioassays

Single dose bioassays used in selection experiments were performed by preparing a stock solution of chlorantraniliprole diluted in RO water at a concentration known to induce 99.9% mortality in the susceptible strain. This, the discriminating concentration, was pre-determined from dose response bioassays described below and established as 0.25 µg of chlorantraniliprole per ml of diet. An aliquot of chorantraniliprole solution required to prepare the discriminating dose was added to 1 L of diet and incorporated using a stick blender to produce a homogenous mixture. The insecticide incorporated diet was then dispensed into 45-well bioassay trays (Tacca Plastics, Moorebank, NSW, Australia). Late second or early third larval instars were placed on treated diet in trays (one larva per well) then covered with heat-sealed perforated lids (Unipac Solutions, Albion Park, NSW, Australia). Untreated diet was used as the control.

Resistance levels in the laboratory reference strain KSL, selected strain BM28x, and various crosses of these two strains were determined by performing dose-response bioassays on artificial diet into which formulated chlorantraniliprole was incorporated. A stock solution of insecticide was diluted with RO water to produce six or seven two-fold serial dilutions with the range of concentrations expected to produce between 0% and 100% mortality in *S. litura* larvae. Each serial dilution was added to 200 ml of artificial diet and vigorously shaken by hand for 30 s to ensure thorough mixing.

The diet incorporation bioassay method was also used to measure cross-resistance of the chlorantraniliprole resistant strain to two other diamide insecticides (cyantraniliprole and tetraniliprole), a meta-diamide insecticide (broflanilide) as well as to emamectin benzoate, indoxacarb, spinetoram and methoxyfenozide. Bioassays to determine sensitivity to the Vip3A toxin in *S. litura* was performed by applying 50 µl aliquots of serially diluted Vip3A solution to the surface of diet contained in 45-well trays and distributing it evenly to ensure that the entire area of the diet was covered with insecticide. Trays were air dried for approx. 30 min before a single unfed *S. litura* neonate was transferred to each well. RO water was used as the control.

Bioassays to test for metabolic resistance were performed using a two-step process^43^ and involved exposing 90 larvae from the resistant strain (G_6_) to the discriminating concentration of chlorantraniliprole prepared using the single-dose diet incorporation bioassay method described, above. After larvae had fed on insecticide incorporated diet for three days, those within a weight range of 30–40 mg were tested by topical application using 1 µl of acetone/synergist solution applied to the dorsal thorax by a 50 µl micro-syringe in a repeating dispenser (Hamilton Company, Reno, NV, USA). Synergist only solutions at a concentration of 50 µg/µl were used as the control.

All bioassays were replicated three times with individual treatments (insecticide concentrations) of each replicate containing a minimum of 20 individual larvae. Bioassays were kept under the same laboratory conditions used for larval rearing described above and were assessed for morality at seven days and considered dead if unable to move in a coordinated manner when prodded.

### Inheritance of chlorantraniliprole resistance in the BM28x strain

**Fig. 1:**
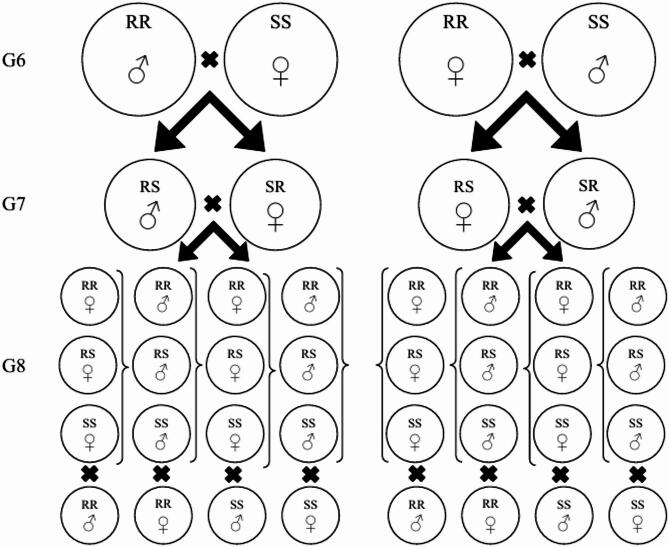
Schematic diagram of crossing and backcrossing process for generating hybrid strains between the resistant strain (BM28x) and susceptible strain (KSL) for determining genetic dominance in G_6_ and recombinants for testing monogenic inheritance in G_7_.

Genetics of resistance was investigated by performing dose-response bioassays on F_1_ progeny from reciprocal crosses between the laboratory reference strain KSL, and the chlorantraniliprole resistant strain BM28x during generations 6, 7 and 8. The process of hybridisation and backcrossing is summarised in Fig. 1 and follows the procedure of Mahon et al.^44^. For the crosses, 360 pupae of each strain were sorted by gender. Six replicates were established: three containing 60 female pupae from the Kununurra strain and 60 male pupae from the BM28x strain, and three containing 60 male pupae from the KLS strain and 60 female pupae from the BM28x strain. The F_1_ progeny from these reciprocal crosses were used to calculate degree of genetic dominance at the median lethal concentration (LC_50_) (*D*_LC_)^45^ to produce values ranging from 0 (completely recessive) to 1 (completely dominant). The LC_50_ values were compared with those from the resistant and laboratory strain tested simultaneously with F_1_ hybrid strains to minimise operator-induced variation. Data from these tests of dominance were also used to evaluate whether resistance was inherited as an autosomal or sex-linked trait.

A test for monogenic inheritance was performed by comparing observed and expected mortalities from a series of reciprocal backcross at single insecticide doses spanning a range of 14 concentrations from 0.0078125 to 64 µg of chlorantraniliprole/ml of diet. Goodness-of-fit (χ^2^) between expected and observed outcomes from bioassays was evaluated in each of two backcrossing events (i.e., F_1_ × KLS reference strain and F_1_ × BM28x resistant strain) with expected values calculated according to the methods of Sokal and Rohlf^46^. Progeny from each of the F_1_ backcross strains was bioassayed synchronously to minimise operator-induced variation. The number of loci associated with resistance was then estimated using the method described, below^47^.

Based on our observations of complete larval survival in the BM28x strain at the discriminating dose of chlorantraniliprole (see ‘Results’) we assumed this strain was homozygous for resistance. Under this scenario, the laboratory KLS strain would consist of only SS genotypes, the resistant BM28x would consist of only RR genotypes and the hybrid F_1_ progeny would consist of RS genotypes. Hence, the backcross of F_1_ and KLS would result in progeny that were 50% SS and 50% RS, and the backcross of F_1_ and BM28x would result in progeny that were 50% RR and 50% RS. The monogenic model was tested by calculating the average probability of mortality for SS, RS and RR individuals (Yx) to each single dose in each of two backcross scenarios^47^:

$${\text{Yx}} = {\text{0}}{\text{.5}} \times \left( {{\text{WF}}_{1} + {\text{WSS}}} \right)\;{\text{and}}\;{\text{Yx}} = 0.5 \times \left( {{\text{WF}}_{1} + {\text{WRR}}} \right)$$

where WF_1_ was the observed proportional mortality for pooled F_1_ progeny of reciprocal crosses between the resistant strain BM28x and the laboratory KLS strain, WSS was the proportional mortality of the laboratory KLS strain, and WRR was the proportional mortality of the resistant BM28x strain. While bioassay results from both backcrosses are presented herein, the backcross of F_1_ and BM28x strain are described in detail because the differences between the F_1_ and the resistant strain were more pronounced than the differences between the F_1_ and the laboratory strain, effectively increasing the level of discrimination between the number of segregating genetic factors^47^.

### Data analysis

The LC_50_ values along with associated 95% fiducial limits (FL) and slope values were calculated using a probit analysis program^48^ based on previously developed source codes^49^. Bioassay data were adjusted for control mortality^50^. Resistance ratios (RR) were determined by dividing the LC_50_ of the resistant strain by the LC_50_ of the laboratory reference strain. The lethal concentration ratio (LCR) test was used to determine significant differences in insecticide toxicity between resistant and susceptible populations of *S. litura*^51^. The proportion of survivors from discriminating dose bioassays relative to the proportion of survivors that would be expected under a scenario where the allele for resistance was recessive was compared using a χ^2^ goodness-of-fit test (IBM Corp. Released 2013. IBM SPSS Statistics for Windows, Version 22.0. Armonk, NY: IBM Corp.). Figures were produced using GraphPad Prism version 8.4.3 (GraphPad Software, Boston, Massachusetts, USA, www.graphpad.com.).

## Results

### Establishment of a discriminating dose of chlorantraniliprole

Baseline susceptibility of chlorantraniliprole in *S. litura* was established from a series of bioassays performed on the laboratory reference strain KSL which had been maintained in continuous laboratory culture for over 35 generations without exposure to insecticides. The susceptibility of the KSL strain was tested on four separate occasions between 2022 and 2024 to confirm consistency of bioassay. The LC_50_ values ranged from 0.023 to 0.028 mg a.i. L^− 1^ of diet with a pooled LC_50_ value of 0.025 mg a.i. L^− 1^ (Table 1). The discriminating dose of chlorantraniliprole was determined from mortality data obtained at the three highest concentrations of the dose range (i.e., 0.0625, 0.12 and 0.25 mg a.i. L^− 1^) and the LC_99.9_ response in the most tolerant cohorts of the KSL strain. There was no survival observed at the two highest concentrations of 0.12 and 0.25 mg a.i. L^− 1^. This was generally consistent with the LC_99.9_ value of the most tolerant cohort (0.218 mg a.i. L^− 1^). However, to ensure that the discriminating concentration provided a high degree of confidence for prevention of false positives, the dose was set at 2-fold higher than the minimum effective concentration i.e., 0.25 mg a.i. L^− 1^ of chlorantraniliprole. This was hereafter considered to represent baseline susceptibility in Australian *S. litura* and the discriminating dose of 0.25 mg a.i. L^− 1^ was subsequently used in calculations to determine resistance ratios of the selected strain and various crosses.

**Table 1:** Bioassays to determine baseline susceptibility in five non-synchronous cohorts of the laboratory reference *S. litura* strain (KSL).

Month and year of bioassay	LC_50_ [mg a.i. L^− 1^] (95% FL)	LC_99.9_ [mg a.i. L^− 1^] (95% FL)	*n*	Fit of probit line			% Mortality at candidate DD^†^ [mg a.i. L^− 1^] (*n*)		
				Slope ± SE	Χ ^2^ (df)	*P*	0.0625	0.125	0.25
July 2022	0.028 (0.025, 0.032)	0.175 (0.116, 0.334)	299	4.50 ± 0.51	4.53 (2)	0.104	90.0 (60)	100 (60)	100 (60)
November 2022	0.023 (0.020, 0.026)	0.155 (0.108, 0.277)	284	3.74 ± 0.47	2.63 (2)	0.268	91.7 (60)	100 (60)	100 (60)
February 2024	0.024 (0.015, 0.036)	0.218 (0.096, 2.643)	360	3.20 ± 0.53	9.18 (3)	0.027	96.7 (60)	100 (60)	100 (60)
June 2024	0.025 (0.022, 0.027)	0.103 (0.079, 0.153)	360	4.98 ± 0.53	0.90 (3)	0.825	96.7 (60)	100 (60)	100 (60)
Pooled	0.025 (0.023, 0.026)	0.156 (0.131, 0.192)	1303	3.86 ± 0.20	4.41 (3)	0.220	93.8 (240)	100 (240)	100 (240)

^†^Discriminating dose.

### Establishment of chlorantraniliprole resistance

Table 2 shows the sequence of events involved in the establishment of the BM28x strain of *S. litura* in laboratory culture. We considered the field collected larval generation was G_0_ and the first two generations of the population (G_1_ and G_2_) were maintained in the laboratory without exposure to insecticide to stabilise the population in preparation for resistance testing. In the G_3_, a preliminary screening bioassay of the *S. litura* population was performed using the discriminating dose of chlorantraniliprole (0.25 mg a.i. L^− 1^) and showed 100% survival in the putative resistant strain, with growth and development of treated larvae similar to that observed in the untreated control larvae (*n* = 180). In contrast, there was 100% larval mortality of the laboratory reference strain KSL (*n* = 300) when exposed to the discriminating dose of chlorantraniliprole (Table 3). A subsequent dose-response bioassay performed in the G_3_ showed this field strain (now designated the BM28x strain) had a resistance ratio of 1078-fold (Table 3). To reduce the risk of inbreeding depression in the BM28x strain, a reciprocal outcross to the KSL strain was performed in the G_3_ followed by reselection at the discriminating dose of chlorantraniliprole in the G_5_ because it was shown the G_4_ was 100% susceptible to chlorantraniliprole and was consistent with a recessive model of inheritance in the BM28x strain (see below). Bioassay of progeny from self-mated F_1_ reciprocal crosses (G_5_) were presumed to be heterozygous for resistance and showed approx. 25% survival at the discriminating dose (Table 3). The surviving individuals were considered to be homozygous for resistance and subsequently used in further quantitative genetic analyses described below.

**Table 2:** Sequence of generational events used to produce strains for characterisation of chlorantraniliprole resistance in the BM28x strain of *S. litura.*

BM28x Generation	Field / laboratory process
G_0_	Field larvae collected from cow pea fields in northwest WA, Australia.
G_1_	Generation maintained in culture.
G_2_	Generation maintained in culture.
G_3_	Dose-response and discriminating dose bioassays. Survivors of discriminating dose (BM28x) outcrossed to KSL.
G_4_	Maintained in culture and discriminating dose bioassay of F_1_ (BM28x × KSL).
G_5_	Discriminating dose bioassays of F_2_ progeny. Survivors used to establish a homozygous resistant colony.
G_6_	BM28x colony selected at the discriminating dose. BM28x survivors of the discriminating dose outcrossed to KSL.
G_7_	Dose-response bioassay of F_1_ (BM28x × KSL). Reciprocal backcross of F_1_ progeny to either BM28x or KSL (Fig. 1).
G_8_	Dose-response bioassay F_1_ backcross strains.

**Table 3:** Responses to chlorantraniliprole in the resistant strain (BM28x), susceptible strain (KSL) and F_1_ progeny of reciprocal crosses.

Strain/cross	LC_50_ [mg a.i. L^− 1^] (95% FL)	*n*	Fit of probit line			RR^‡^	D_LC_	% survival at DD^†^ (*n*)
			Slope ± SE	Χ ^2^(df)	*P*			
KSL (susceptible)	0.025 (0.022, 0.027)	360	4.98 ± 0.53	0.90 (3)	0.825	–	–	0 (300)
(G_3_) BM28x (resistant)	26.9 (21.3, 38.1)	258	2.09 ± 0.32	3.73 (2)	0.155	1078	–	100 (180)
(G_4_) F_1_: KSL♀ × BM28x♂	NT							0 (90)
(G_4_) F_1_: KSL♂ × BM28x♀	NT							0 (90)
(G_5_) (KSL♀ × BM28x♂) × (KSL♀ × BM28x♂)	NT							24.4 (1436)
(G_5_) (KSL♂ × BM28x♀) × (KSL♂ × BM28x♀)	NT							24.9 (1799)
(G_5_) Pooled crosses	NT							24.7 (3235)
(G_6_) Selected at discriminating dose	NT							100 (540)
(G_6_) Selected at discriminating dose + PBO	NT							100 (90)
(G_6_) Selected at discriminating dose + TPP	NT							100 (90)
(G_6_) Selected at discriminating dose + DEM	NT							100 (90)
(G_7_) F_1_: KSL♀ × BM28x♂	0.099 (0.087, 0.113)	359	3.38 ± 0.32	0.71 (3)	0.871	4.0	0.197	0 (60)
(G_7_) F_1_: KSL♂ × BM28x♀	0.084 (0.073, 0.095)	360	3.48 ± 0.34	2.05 (3)	0.562	3.4	0.174	0 (60)
(G_7_) F_1_: Pooled crosses	0.091 (0.083, 0.100)	719	3.43 ± 0.23	1.55 (3)	0.671	3.6	0.185	0 (120)
(G_8_) Backcross F_1_ × KSL	0.037 (0.034, 0.039)	1911	3.18 ± 0.14	5.54 (5)	0.354	1.5	–	0 (240)
(G_8_) Backcross F_1_ × BM28x	0.804 (0.390, 1.495)	2605	0.81 ± 0.12	88.46 (8)	0.0001	32.2	–	52.9 (237)

Synergists tested in G_6_ were piperonyl butoxide (PBO), Triphenyl phosphate (TPP), and diethyl maleate (DEM) topically applied at a concentration of 50 µg/µl.

*NT* Not tested.

^†^Discriminatingconcentration.

^‡^Resistance ratio: LC_50_ of resistant strain / LC_50_ of laboratory susceptible strain.

### Estimates of genetic dominance in the BM28x strain

Discriminating dose bioassays of the G_6_ generation resulted in 100% survival at the discriminating dose (*n* = 540). At this point it was assumed that homozygosity in the BM28x population had been restored, and a reciprocal outcross was performed using G_6_ moths from BM28x crossed with moths from the KSL strain to create the G_7_ generation. Bioassays on F_1_ progeny from these crosses show LC_50_ values of 0.099 and 0.084 mg a.i. L^− 1^ in the KSL♀ × BM28x♂ and KSL♂ × BM28x♀ strains, respectively (Table 3). The LC_50_ values from the two reciprocal crosses (G_7_) were similar based on a lethal dose ratio test (LCR = 0.843; 95% CI 0.699–1.0183), suggesting a high likelihood that inheritance of resistance in the BM28x strain was autosomal with no sex linkage or maternal effects. In F_1_ heterozygotes (G_7_), the resistance ratio was 3.6-fold (pooled LC_50_ from the reciprocal crosses was 0.091 µg/ml) and the degree of dominance (*D*_*LC*_) for the pooled reciprocal crosses was 0.185 (Table 3).

Under a model of recessive inheritance, the predicted gene frequency in the G_4_ was 0.5 and the rate of survival at the discriminating concentration of chlorantraniliprole in the G_5_ (progeny resulting from self-mating between heterozygotes) was expected to be 25%. Our results demonstrate inheritance of chlorantraniliprole resistance in the BM28x strain was strongly recessive based on the observation of complete mortality in F_1_ progeny from crosses of BM28x and KSL strains (G_4_ generation) (Table 3) and the observed proportion of larvae able to survive the discriminating concentration, which was not statistically different to the level of survival expected if resistance was recessive (proportion observed = 0.247, χ^2^ = 0.191, *P* = 0.662).

Table 4 shows the estimates of effective dominance (*D*_ML_) from bioassays of the resistant, reference and F_1_ strains spanning 14 concentrations of chlorantraniliprole. These results indicate that dominance was dose-dependent and decreased as insecticide concentration increased in the F_1_ strain. However, resistance was almost completely recessive at the discriminating concentration of 0.25 mg a.i. L^− 1^ (D_*ML*_ = 0.07) (Table 4).

**Table 4:** Dominance of chlorantraniliprole resistance in *S. litura* as a function of chlorantraniliprole concentration.

Concentration [mg a.i. L^− 1^]	Survival (%)			Effective dominance (D_ML_)
	KSL Susceptible	F_1_	BM28x Resistant	
0.0078125	100	100		1
0.015625	83.3	99.2		0.95
0.03125	28.3	94.2		0.92
0.0625	3.3	69.2		0.68
0.125	0.0	35.3		0.35
0.25		6.7		0.07
0.5		0.0		0
1				
2			100.0	
4			84.5	
8			62.7	
16			50.8	
32			10.2	
64			0.0	

### A test for monogenic inheritance of chlorantraniliprole resistance

**Fig. 2:**
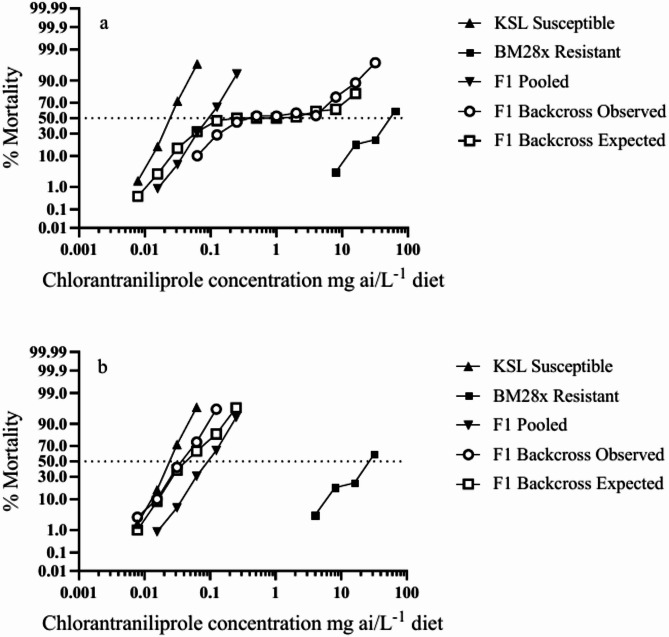
Response of chlorantraniliprole in *S. litura* from laboratory and selected strains, F_1_ progeny (pooled data from reciprocal crosses) and backcross progeny (F_1_ × resistant BM28x strain, a) and (F_1_ × laboratory KSL strain, b). The observed F_1_ backcross curve shows mortality data at each concentration. The expected F_1_ backcross curve shows predicted values of mortality calculated from a single locus model according to methods of Tabashnik (1991).

The levels of observed and expected mortality from χ^2^ analysis of the F_1_ × BM28x backcross were not significantly different (*P* > 0.05) at five of the ten intermediate concentrations tested (0.5–8.0 mg a.i. L^− 1^). However, significant deviations (*P* < 0.0001) were found at another the five concentrations at the extreme lower and upper ends of the dose response curve, these being the three lowest doses (0.0625, 0.125 and 0.25 mg a.i. L^− 1^) and the two highest doses (16 and 32 mg a.i. L^− 1^) (Table 5) suggesting there may be a multifactorial mode of inheritance in the BM28x strain. However, there was a strong inflection point at the median lethal concentration (Fig. 2a) which is consistent with a hypothesis of increased heterogeneity in the F_1_ × BM28x backcross strain compared with the parental BM28x and F_1_ strains. Further evidence of increased genetic variation can be seen in the pattern of slopes associated with the dose mortality response in progeny from the F_1_ × BM28x backcross which shows a slope value of 0.81 ± 0.12. This was 4.8-fold lower than the slope of the laboratory KSL strain (3.86 ± 0.20), 2.5-fold lower than the slope for the resistant strain (2.09 ± 0.32), and 4.2-fold lower than the slope from the pooled bioassays of the F_1_ strains (3.43 ± 0.23) (Table 3; Fig. 2a). On the other hand, the slope value of the F_1_ × KSL backcross (3.18 ± 0.14) was similar to the pooled F_1_ strains and the KSL strain (Table 3; Fig. 2b). The accumulated evidence of increased genetic variation in the F_1_ × BM28x backcross compared with the parental and F_1_ strains may indicate that resistance is controlled by either a single major genetic factor or multiple closely linked loci that segregate together.

**Table 5:** Direct test of monogenic inheritance for resistance to chlorantraniliprole by comparing expected and observed mortality of the backcross (F_1_ × BM28x) of *S. litura*.

Concentration [mg a.i. L^− 1^]	No. of larvae tested	Observed mortality (proportion)	Expected mortality (Y_x_)	χ^2^ (df = 1)	*P*
0.0625	220	22 (0.10)	0.15	43.6	< 0.0001
0.125	238	68 (0.29)	0.32	49.7	< 0.0001
0.25	240	107 (0.45)	0.47	31.3	< 0.0001
0.5	236	126 (0.53)	0.50	2.79	0.0948
1	237	125 (0.53)	0.50	0.98	0.3216
2	240	137 (0.57)	0.50	0.81	0.3674
4	240	129 (0.54)	0.52	2.81	0.0935
8	239	182 (0.76)	0.59	3.30	0.0694
16	238	211 (0.89)	0.62	21.7	< 0.0001
32	238	231 (0.97)	0.80	10.8	0.001

### Insecticide cross resistance

Chlorantraniliprole resistance in the BM28x strain conferred strong cross-resistance to the two other Group 28 insecticides, cyantraniliprole and tetraniliprole, where resistance ratios were 262-fold and 591-fold, respectively (Table 6). Although cyantraniliprole was approx. 3-fold less toxic on the KSL strain than chlorantraniliprole there was no significant difference in the toxicity of these two diamides in the BM28x strain (LCR = 0.748; 95% CI 0.551–1.016). Similarly, tetraniliprole was approx. 2-fold less toxic than chlorantraniliprole in the KSL strain but not signifiantly different to chlorantraniliprole in the BM28x strain (LCR = 1.21; 95% CI 0.888–1.639).

**Table 6:** Resistance levels in the chlorantraniliprole-resistant strain (BM28x) and laboratory strain (KSL) to conventional insecticides.

Insecticide	Population	LC_50_ [mg a.i. L^− 1^] (95%FL)	*n*	Fit of probit line			RR^‡^
				Slope ± SE	*χ*^2^ (df)	*P*	
Chlorantraniliprole	KSL	0.025 (0.022, 0.027)	360	4.98 ± 0.53	0.90 (3)	0.825	–
	BM28x	26.9 (21.4, 38.1)	258	2.09 ± 0.32	3.73 (2)	0.155	1078
Cyantraniliprole	KSL	0.077 (0.069, 0.087)	338	4.46 ± 0.46	3.70 (3)	0.296	262
	BM28x	20.2 (17.7, 23.0)	360	3.39 ± 0.32	4.86 (3)	0.182	
Tetraniliprole	KSL	0.055 (0.049, 0.061)	259	5.40 ± 0.61	0.50 (2)	0.779	591
	BM28x	32.5 (28.5, 37.1)	317	3.44 ± 0.32	2.06 (3)	0.560	
Broflanilide	KSL	0.828 (0.751, 0.912)	296	6.54 ± 0.81	2.43 (2)	0.297	1.3
	BM28x	1.084 (0.971, 1.203)	428	4.34 ± 0.41	6.46 (3)	0.091	
Indoxacarb	KSL	0.254 (0.119, 0.529)	259	3.27 ± 0.67	7.47 (2)	0.024	3.3
	BM28x	0.847 (0.713, 1.004)	497	2.04 ± 0.15	7.16 (6)	0.306	
Emamectin benzoate	KSL	0.021 (0.019, 0.023)	300	5.80 ± 0.67	3.02 (2)	0.221	2.8
	BM28x	0.059 (0.022, 0.103)	420	1.38 ± 0.26	10.78 (4)	0.029	
Spinetoram	KSL	0.814 (0.699, 0.938)	285	3.12 ± 0.34	4.93 (2)	0.085	2.3
	BM28x	1.909 (1.639, 2.221)	405	2.59 ± 0.22	6.68 (4)	0.142	
Methoxyfenozide	KSL	0.328 (0.298, 0.362)	365	6.47 ± 0.79	0.45 (3)	0.930	1.6
	BM28x	0.516 (0.463, 0.575)	365	5.09 ± 0.55	1.37 (3)	0.713	
Vip3A	KSL	0.363 (0.293, 0.445)	493	1.53 ± 0.12	8.43 (6)	0.208	1.2
	BM28x	0.444 (0.335, 0.582)	495	1.06 ± 0.10	1.90 (6)	0.929	

^‡^Resistance ratio: LC_50_ of resistant strain / LC_50_ of laboratory susceptible strain.

Broflanilide was approx. 33-fold less toxic than chlorantraniliprole on the KSL strain with an LC_50_ value of 0.828 mg a.i. L^− 1^. However, there was very low cross-resistance to broflanilide in the BM28x strain (LC_50_ = 1.084 mg a.i. L^− 1^) (Table 6). Likewise, there were very low levels of cross-resistance to indoxacarb (3.3-fold), emamectin benzoate (2.8-fold), spinetoram (2.3-fold) and methoxyfenozide (1.6-fold) (Table 6). There was also no evidence of reduced sensitivity to the Vip3A toxin with similar levels of toxicity in both the resistant and susceptible strains (Table 6).

### Synergism of resistance in the BM28x strain

There was no mortality in the BM28x strain (G_6_) when larvae were exposed to the discriminating dose of chlorantraniliprole. Topical application of PBO, TPP or DEM to resistant larvae pre-treated with chlorantraniliprole at the discriminating dose did not increase mortality (Table 3), indicating resistance is not mediated by metabolic enzymes.

## Discussion

Over the last 15 years diamide insecticides have been relied upon in agricultural production for managing a range of lepidopteran pests. However, the popularity of these products has increased the intensity of selection pressure and led to a global escalation of resistance development in target species^13^. *Spodoptera litura* is a serious pest which has demonstrated a track record for evolving field resistance to diamide insecticides in China since 2008^36,52,53^. Despite evidence that incipient diamide resistance in southern China had reverted after 2015^36,52^, a recent study reported 131-fold target site resistance to chlorantraniliprole in *S. litura* populations from the Chinese province of Fujian was associated with the RyR mutation I4728M (corresponding to RyR I4790M in *P. xylostella*)^10^. In another recent study, populations of *S. litura* from eastern China showed high resistance to chlorantraniliprole (77-fold), with cross-resistance to the other diamide insecticides flubendiamide (25-fold) and cyantraniliprole (28-fold)^54^.

Here we report the first case of diamide resistance in Australian *S. litura*, and our results show that the magnitude of resistance in the BM28x strain of *S. litura* from northwestern Australia was significantly higher than levels reported in *S. litura* from China, with a resistance ratio of 1078-fold in the BM28x strain compared with the laboratory reference strain KSL. This high level of chlorantraniliprole insensitivity is consistent with that previously reported in populations with target site resistance conferred by amino acid polymorphisms on the RyR channel. For example, the I4790M mutation^18^ and G4946E^55^ conferred ≥ 2000-fold resistance in *P. xylostella*. The high level of resistance in the BM28x strain is also consistent with anecdotal evidence from areas of intensive horticultural production in northwestern regions of Australia which have experienced complete field failures of chlorantraniliprole applied to *S. litura* populations.

The rate of increase in resistance allele frequency is strongly influenced by the degree of genetic dominance of resistance alleles. If resistance is controlled by dominant genes, resistance frequency is expected to increase rapidly through survival of heterozygous genotypes. Conversely, when resistance is controlled by recessive genes, the rate of increase is expected to occur more slowly because the proportion of functionally susceptible genotypes in the population is higher than if resistance was dominant^56^. Studies that have investigated inheritance of diamide resistance have shown that in cases where resistance is mediated by point mutations at the RyR, resistance is largely recessive and autosomal. For example, resistance was almost completely recessive in a strain of *P. xylostella* with > 10,000-fold resistance to flubendiamide conferred by the G4946E mutation^15^. Similarly, a case of > 1000-fold G4946E mediated resistance to flubendiamide in *S. exigua* was found to be completely recessive^57^.

As in other species such as *P. xylostella*^15^, *S. exigua*^57^, and *C. suppressalis*^17^ where resistance is mediated by point mutations at the RyR, our results show that diamide resistance in Australian *S. litura* is inherited as autosomal and almost completely recessive trait, with a degree of dominance of 0.185. However, we also do not rule out the possibility that F_1_ progeny from crosses of BM28x and KSL may have had a measure of hybrid vigour that was expressed as reduced susceptibility to chlorantraniliprole relative to the KSL strain. Results from backcrosses of the F_1_ progeny with the resistant parental strain, combined with a high magnitude of resistance in the BM28x strain (> 1000-fold) provides further evidence that diamide resistance this population of *S. litura* is conferred either by a single genetic factor or a small group of closely linked genes that segregate together.

Although most documented cases of diamide resistance in lepidopteran species are mediated primarily by target site resistance mechanisms, metabolic resistance appears to play a secondary role in terms of contributing to field-level control failures of diamide insecticides and may not produce practical resistance by themselves^13^. Diamide resistance mediated by metabolic detoxification systems also appears to be less straightforward than target site resistance and can have diverse pattens of inheritance. For example, chlorantraniliprole resistance associated with upregulation of detoxification enzymes in *P. xylostella* were shown to be autosomal and multifactorial with incompletely recessive inheritance^58,59^. Likewise, metabolically mediated chlorantraniliprole resistance in *S. litura* from China was autosomal and incompletely recessive^54^, while diamide resistance in *S. frugiperda* from Brazil displayed varying inheritance trends and biochemical profiles for different insecticides^38^. Results from our present study of chlorantraniliprole resistance in the BM28x strain of *S. litura* show that metabolic resistance does not play a role in diamide insensitivity, and a target site polymorphism may be the likely causal mechanism. We are currently performing genotype-by-sequencing analysis to determine molecular basis of resistance in the BM28x strain.

The confirmation by quantitative genetic analysis that chlorantraniliprole resistance in the BM28x strain was recessive and that the progenitor population of this strain was already homozygous for chlorantraniliprole resistance in the field generation (G_0_) at the time it was sampled is of serious concern because it demonstrates that *S. litura* in intensively managed horticultural regions of northwest WA likely contained a significant proportion of heterozygotes for some considerable period of time before evidence emerged that diamide insecticides were unable to provide field control. Further, despite recessive inheritance of resistance, ample mating between heterozygotes had occurred which was able to drive resistance to fixation.

However, our cross-resistance study of the BM28x strain showed that although significant resistance extended to the two diamide insecticides cyantraniliprole and tetraniliprole, there was no major cross resistance to broflanilide, emamectin benzoate, spinetoram, methoxyfenozide or the Vip3A protein produced by transgenic cotton widely used in Australia. Hence, alternative MoA insecticides should provide a rotational framework for effective management of resistance, despite the reduced efficacy of the diamide insecticides^60,61^.

The effectiveness of rotational strategies could be further enhanced if insecticide resistance mechanisms are associated with fitness costs, as alterations to the functionality of receptor proteins (target sites) often have deleterious effects on the overall fitness of resistant insects which may place them at a competitive disadvantage compared to susceptible insects. For example, lower reproductive potential was found in a cyantraniliprole-selected laboratory strain of *P. xylostella*^62^. However, even if fitness costs are thought to be associated with resistance alleles, they may be overestimated if fitness comparisons are not made between isogenic resistant and susceptible strains. Alternatively, they may not be of sufficient magnitude to cause a decrease in resistance frequency in the absence of selection pressure (e.g^63^). There may even be higher fitness values associated with resistance (e.g^64^) due to the evolution of compensatory modifier genes^65^. We are currently performing a stability analysis using near isogenic lines of chlorantraniliprole resistant and susceptible *S. litura* to determine whether a fitness cost is associated with diamide resistance identified in this study. This information combined with the results from the present study will be important for informing appropriate resistance management strategies for ongoing and sustainable management of *S. litura*.

## Data Availability

All relevant data are within the paper. Datasets generated during and/or analysed during the current study are available from the corresponding author on request and with approval from the funding agency the Australian Cotton Research and Develpment Corporation (CRDC).
